# Comparison between dexmedetomidine and propofol on outcomes after coronary artery bypass graft surgery: a retrospective study

**DOI:** 10.1186/s12871-022-01589-6

**Published:** 2022-02-19

**Authors:** Jie Hu, Bingfeng Lv, Raha West, Xingpeng Chen, Yali Yan, Chen Pac Soo, Daqing Ma

**Affiliations:** 1 Department of Anesthesiology, Luoyang Central Hospital Affiliated to Zhengzhou University, Luoyang, Henan China; 2 Division of Anaesthetics, Pain Medicine and Intensive Care, Department of Surgery and Cancer, Faculty of Medicine, Imperial College London, Chelsea and Westminster Hospital, London, UK; 3 Department of cardiovascular surgery, Luoyang Central Hospital Affiliated to Zhengzhou University, Luoyang, Henan China; 4 Information Center, Luoyang Central Hospital Affiliated to Zhengzhou University, Luoyang, Henan China; 5 Department of Anesthesiology, Wycombe General Hospital, Queen Alexandra Road, High Wycombe, Buckinghamshire HP11 2TT UK

**Keywords:** Dexmedetomidine, Propofol, CABG, Pulmonary complications, Surgical time

## Abstract

**Background:**

Dexmedetomidine (DEX) has a pharmacological profile that should allow rapid recovery and prevent undesirable outcomes such as pulmonary complications.

**Methods:**

This large retrospective study compared the beneficial effects of perioperative infusion of DEX with propofol on the postoperative outcome after coronary artery bypass graft surgery. We reviewed patients’ medical notes at Luoyang Central Hospital from 1st January 2012 to 31st December 2019. All continuous variables, if normally distributed, were presented as mean ± SD; Otherwise, the non-normally distributed data and categorical data were presented as median (25-75 IQR) or number (percentage). The Mann-Whitney U test and Chi-square test were used to evaluate the difference of variables between the DEX and propofol groups. Multivariate logistic regression analysis was performed on the main related and differential factors in the perioperative period.

**Results:**

A total of 1388 patients were included in the study; of those, 557 patients received propofol infusion, and 831 patients received dexmedetomidine. DEX significantly reduced postoperative pulmonary complications compared with propofol, 7.82% *vs* 13.29%; *P* < 0.01, respectively. When compared with propofol, DEX significantly shortened the duration of mechanical lung ventilation, 18 (13,25) hours vs 21 (16,37) hours; *P* < 0.001, the length of stay in the intensive care unit, 51 (42,90) vs 59 (46,94.5) hours; *P* = 0.001 and hospital stay, 20 (17,24) vs 22 (17,28) days; *P* < 0.001, respectively. The incidences of postoperative wound dehiscence and infection were significantly reduced with DEX compared with propofol groups, 2.53% vs 6.64%; *P* *<* 0.001, respectively. Interestingly, patients receiving DEX had significantly shorter surgical time compared to propofol; 275 (240,310) vs 280 (250,320) minutes respectively (*P* = 0.005) and less estimated blood loss (*P* = 0.001).

**Conclusion:**

Perioperative infusion of dexmedetomidine improved the desirable outcomes in patients who had coronary artery bypass graft surgery compared with propofol.

## Introduction

The optimum intraoperative anaesthetic agent for cardiac surgery should allow the patients to recover rapidly and prevent undesirable outcomes such as pulmonary complications, prolonged mechanical lung ventilation, and prolonged stay in the intensive care unit (ICU). Prolonged mechanical ventilation and ICU stay are associated with high morbidity and mortality rates following cardiac surgery [1, 2]. Anaesthetic techniques and agents used during surgery to accelerate weaning from mechanical lung ventilation and patient’s recovery are essential for fast-track cardiac anaesthesia and are increasingly being adopted.

Dexmedetomidine (DEX) is a highly selective short-acting α_2_-adrenoceptor agonist with properties including sedative, analgesic, anxiolytic, opioid and anaesthetic sparing effects [3]. DEX has minimal impact on respiratory depression, improves oxygenation and lung compliance, and reduces postoperative pulmonary complications [4, 5]. DEX also alleviates perioperative stress, inflammatory and immune response leading to an excellent postoperative recovery [6]. Perioperative use of DEX as an anaesthetic adjunct and postoperative sedation was reported to reduce the time spent on mechanical ventilation, improve 30 days mortality, shorten ICU and hospital stay, and decrease postoperative complications, including the incidence of pulmonary complications and delirium and acute kidney injury [7, 8].

Several studies demonstrated the benefit of DEX infusion in providing haemodynamic stability during cardiac surgery [9–11]. Meta-analysis studies on the use of DEX during cardiac surgery also showed a reduction in the risk of atrial fibrillation, ventricular tachycardia and cardiac arrest [7, 12]. The potential impact of the haemodynamic stability provided by DEX during cardiac surgery on intraoperative outcomes is still limited.

In this study, we analysed our patients’ data retrospectively. We investigated the potential benefits of DEX compared to propofol during and after anesthesia and surgery on postoperative outcomes in patients undergoing coronary artery bypass graft surgery (CABG). We also explored the potential benefits of DEX infusion during surgery on intraoperative outcomes such as blood loss, blood transfusion, duration of anaesthetic and surgery, and opiates consumption in those patients.

## Methods

### Study design and setting

This retrospective cohort study was approved by the Ethics committee of Luoyang Central Hospital, Zhengzhou University, Henan, China. Because of its retrospective study nature, the need for informed consent was waived. All methods were performed following the relevant guidelines and regulations. The manuscript was prepared according to the statement on the Strengthening the Reporting of Observational Studies in Epidemiology (STROBE) [13].

### Participants

The inclusion criteria were patients aged 18 and above who received either DEX or propofol infusion during CABG surgery as an adjunct for general anaesthesia and as a postoperative sedative drug until extubation in the ICU. Exclusion criteria were patients receiving DEX and propofol together at any time during the intraoperative and postoperative periods except for an induction dose of propofol at the start of general anaesthesia and patients who had severe comorbidities, including valvular heart disease, infections, and lung, kidney and liver dysfunction.

### Perioperative management

After the patients had received premedication of 10 mg morphine and 0.3 mg scopolamine by intramuscular injection, they were anesthetised with etomidate (0.3 mg/kg), sufentanil (0.8 μg/kg) and atracurium (0.2 mg/kg), their tracheas were intubated, and their lungs were mechanically ventilated (I/E = 1:1.5; VT = 6-8 ml/kg; 10-12/min). Anesthesia was maintained with infusions of propofol or DEX initially at 2-12 mg/kg/hr. or 1.0 μg/kg/hr., respectively, and reduced thereafter to DEX 0.3 μg/kg/hr. All patients received 1-2% sevoflurane, sufentanil (0.6-0.8 μg/kg) and atracurium (0.1 mg/kg) when required during surgery. Patients received routine monitoring, including invasive arterial pressure, Bispectral Index (BIS) (Covidien, USA) value, body temperature, pulse oximetry and ECG. Pulmonary artery pressure, cardiac index and cardiac output were continuously monitored *via* a Swan-Ganz catheter (Vigileo II, Edwards, Irvine, USA).

The patients underwent median sternotomy; the left internal mammary artery and part of the saphenous vein were harvested at normal body temperature. Heparin was administered intravenously to adjust the activated clotting time readings within the appropriate range. CABG was performed on a beating heart with off-pump surgery as a routine procedure. However, if the mean pulmonary artery pressure was higher than 50% of the mean arterial pressure and the patient was haemodynamically unstable when surgeons attempted to elevate the heart for surgery, a heart beating on-pump surgery was performed using partial assistance from the cardiopulmonary bypass (CPB). The patient was connected to the CPB circuit to maintain circulation, and the coronary artery bypass grafting was completed under CPB assistance without cardiac arrest. Methoxamine was used to increase peripheral vascular resistance when raising the patient’s heart during off-pump CABG, with volume control to maintain circulatory stability and reduce myocardial oxygen consumption. Because it is necessary to control the patient’s heart rate during off-pump CABG, only methoxamine was used without other positive inotropic drugs, for example, norepinephrine, which could excite the β receptor.

### Outcomes

The primary outcome measured was pulmonary complication following CABG between patients who received DEX or propofol. This was defined as any pre-defined pulmonary complications following surgery, including respiratory infection, respiratory failure, pleural effusion, atelectasis, pneumothorax, bronchospasm, aspiration pneumonitis, pulmonary oedema, pulmonary embolism, and acute respiratory distress syndrome.

The secondary outcomes include the duration of mechanical lung ventilation, postoperative morbidity, 30-day mortality and length of stay (LOS) in ICU and hospital. We also looked at exploratory outcomes of intraoperative findings such as surgical times and estimated blood loss.

### Data collection

Demographic data such as age, gender, body mass index, comorbidities including cardiac arrhythmia type and alcohol consumption (alcoholism: defined as drinking wine during two meals/day), American Society of Anaesthesiology (ASA) physical status classification and laboratory data of kidney function, blood count and C-reactive protein (CRP) were collected. Intraoperative data included inotropic drugs used, anaesthetic agents, duration of anaesthesia, duration of surgery, intraoperative fluid, urine output, autologous blood transfusion, extracorporeal circulation and intra-aortic balloon pump assistance.

Postoperative data including new-onset cardiac arrhythmia and complications after surgery were also collected as outcome measures. Postoperative pulmonary complications were defined as postoperative pneumonia, hypoxemia (PaO_2_/FiO_2_ ≤ 300 mmHg), postoperative respiratory failure (PaO_2_/FiO_2_ ≤ 200 mmHg and required mechanical ventilation for more than 48 h) and atelectasis and bronchospasm exacerbation of pre-existing chronic lung disease. Surgical bleeding was defined as chest tube drainage exceeding 500 ml per hour or 200 ml per hour for three consecutive hours accompanied by blood volume replacement and hemodynamic instability.

### Statistical analysis

All continuous variables, if normally distributed, were presented as mean ± SD; otherwise, the non-normally distributed data and categorical data were presented as median (25-75 IQR) or number (percentage). First, we compared the clinical characteristics, including demographic data, laboratory data, post-operative data and data during surgery. The Mann-Whitney U test was used to analyze continuous variables. The Chi-square test was used to compare categorical variables to evaluate the difference between the DEX and the propofol group. Based on the statistical comparisons between the two groups, the significant preoperative factors and important perioperative factors related to the postoperative pulmonary complications such as time of surgery, length of stay in the hospital, duration of mechanical ventilation, length of stay in ICU after surgery, DEX use, CPB assistance, diabetes, wound infection, age and BMI were integrated into a multivariate logistic regression model, and the adjusted ORs, 95% CIs, and P values were calculated for each variable. All data were analysed with SPSS 22 (ABS, USA). A P value less than 0.05 was considered to be of statistical significance.

## Results

### Baseline patient demographic and perioperative characteristics

**Fig. 1:**
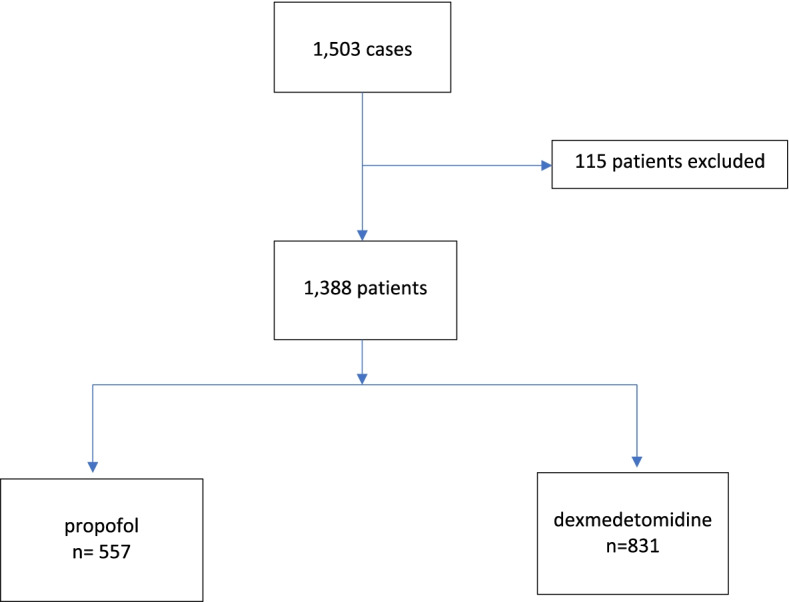
Flow chart of patients included for data analysis

Routinely collected data were captured by the direct care team for patients undergoing CABG at Luoyang Central Hospital from 1st of January 2012 to 31st of December 2019. A total of 1503 patients had CABG during the studied period being carried out by the same team of surgeons and anesthesiologists; 115 of them were excluded based on our exclusion criteria. Data for the study were from the remaining 1388 patients (Fig. 1). Of those, 831 patients received DEX, and 557 received propofol as an anaesthetic adjunct during surgery and postoperative sedation. There were no significant differences between the two groups in terms of age, gender, body mass index, blood type, ASA classification and comorbidities, including stroke, chronic obstructive pulmonary disease, diabetes, hyperlipidaemia, smoking, liver dysfunction, chronic kidney disease and chronic heart failure (Table 1). There were more patients with cardiac arrhythmia comorbidity (6.64% vs 3.37%, *P* = 0.005) and alcoholism (29.44% vs 19.98%, *P* < 0.001) in the propofol compared to the DEX group. Before surgery, there were no differences in kidney function, white blood cells, and neutrophils between the two groups. However, all measurements were significantly raised after surgery in the propofol group compared to DEX (Table 2).

**Table 1:** Demographic data

	Propofol (*n* = 557)	Dexmedetomidine (*n* = 831)	*P* value
Age	62 (56,68)	63 (57,68)	0.515
Gender (male/female)	422/135	615/216	0.461
BMI (kg/m^2^)	24.91(23.29,26.08)	24.61(22.76,26.45)	0.184
Blood type			
A	143 (26.67%)	217 (26.11%)	0.493
B	179 (32.14%)	267 (32.13%)	
O	182 (32.67%)	249 (29.97%)	
AB	53 (9.52%)	98 (11.79%)	
Co-comorbidity			
Cardiac arrhythmia	37 (6.64%)	28 (3.37%)	0.005
Sinus tachycardia	6	4	0.198
Sinus bradycardia	5	4	0.344
Atrial fibrillation	15	10	0.041
Atrial premature beats	7	6	0.369
Atrioventricular block	4	4	0.721
Stroke	101(18.13%)	122 (14.68%)	0.086
COPD	10 (1.79%)	21 (2.53%)	0.366
Diabetes	167 (29.98%)	236 (28.4%)	0.524
Hyperlipidemia	146 (26.21%)	209 (25.15%)	0.657
Smoke	251 (45.06%)	387 (46.57%)	0.581
Alcoholism	164 (29.44%)	166 (19.98%)	<0.001
Liver dysfunction	9 (1.62%)	11 (1.32%)	0.654
Chronic kidney disease	8 (1.44%)	12 (1.44%)	0.990
Chronic heart failure	53 (9.52%)	58 (6.98%)	0.088
ASA classification			
II	26 (4.67%)	32 (3.85%)	0.796
III	528 (93.71%)	795 (95.67%)	
IV	9 (1.62%)	4 (0.48%)	

Data are median (IQR) and patient’s number (%)

**Table 2:** Laboratory data

	Propofol (n = 557)	Dexmedetomidine (*n* = 831)	*P* value
Preoperation			
Urea nitrogen (mmol/l)	3.29(3.05,3.89)	3.47(3.05,3.89)	0.181
Creatinine (μmol/l)	38(31,48)	38(31,48)	0.862
White blood cells (10^X9^/L)	4.08(3.89,4.28)	4.08(3.89,4.29)	0.924
Neutrophiles (10^X9^/L	3.26(3.02,3.89)	3.29(3.02,3.89)	0.318
Postoperation			
Urea nitrogen (24 h, mmol/l)	6.37(6.16,7.07)	6.26(6.14,7.07)	0.003
Creatinine (24 h, μmol/l)	69(61,89)	68(58,89)	<0.001
White blood cells (72 h,10^X9^/L)	10.06(9.37,10.37)	9.27(9.07,9.97)	<0.001
Neutrophiles (72 h,10^X9^/L)	6.48(6.14,7.07)	6.26(6.05,7.03)	<0.001

Data are median (IQR) and patient’s number (%)

### Primary outcomes

Perioperative DEX significantly reduced pulmonary complications collectively, including hypoxemia, atelectasis, pneumonia, bronchospasm, and pleural effusion, with 7.82% total complications in the DEX compared to 13.29% in the propofol group (*P* < 0.01). When broken down into the individual pulmonary complication, although the general trend, except for pleural effusion, pointed towards a better pulmonary outcome for the DEX group, only atelectasis was statistically different with 1.32% incidence in the DEX compared to 2.87% in propofol (*P* = 0.048) (Table 3).

**Table 3:** Postoperative data

	Propofol (*n* = 557)	Dexmedetomidine (*n* = 831)	*P* value
All cause 30-day mortality	12	21	0.655
New onset arrythmia	22 (3.95%)	37 (4.45%)	0.649
Atrial fibrillation	12	9	0.109
Ventricular fibrillation	10	27	0.099
Frequent premature ventricular contractions	0	1	1.000
Pulmonary complications	74 (13.29%)	65 (7.82%)	<0.01
Hypoxemia	23	19	0.056
Atelectasis	16 (2.87%)	11 (1.32%)	0.048
Pneumonia	26	24	0.081
Bronchospasm	5	4	0.344
Pleural effusion	4 (0.72%)	7 (0.84%)	1.000
Upper gastrointestinal bleeding	1 (0.18%)	2 (0.24%)	1.000
Surgical bleeding^a^	8 (1.44%)	5 (0.60%)	0.114
Wound dehiscence or infection	37 (6.64%)	21 (2.53%)	<0.001
Acute kidney injury	4 (0.72%)	6 (0.72%)	1.000
Stroke	2 (0.36%)	3 (0.36%)	1.000
Coronary artery CTA follow up	58 (10.41%)	67 (8.06%)	0.134
Within three months, Bridge vascular root	12	83	
Within three months, Bridge vascular recanalization rate	10/12(83.33%)	77/83(92.77%)	0.586
Within 1 year, Bridge vascular root	17	164	
Within 1 year, Bridge vascular recanalization rate	14/17(82.35%)	147/164(89.63%)	0.613
More than 2 years, Bridge vascular root	173	202	
More than 2 years, Bridge vascular recanalization rate	147/173(84.79%)	184/202(91.09%)	0.066
Length of stay in hospital, days	22 (17,28)	20 (17,24)	<0.001
Duration of mechanical ventilation, hrs	21 (16,37)	18 (13,25)	<0.001
Length of stay in ICU after surgery, hrs	59 (46,94.5)	51 (42,90)	0.001

Surgical bleeding^a^ defined as the chest tube drainage exceeds 500 ml per hour or 200 ml per hour for 3 consecutive hours, with blood volume replacement and hemodynamic instability

Data are median (IQR), patient’s number (%) or mean ± SD; CTA: Computed Tomography Angioplasty

Looking at important perioperative factors that could influence postoperative pulmonary complications, DEX (OR 0.544, *P* = 0.002) and CPB (0.140, *P* < 0.001) were associated with a decrease in postoperative lung complications but diabetes (OR 1.500, *P* = 0.040) and wound infection (OR 3.995, *P* < 0.001) increased the risk of lung complications (Table 4). Preoperative cardiac arrhythmia and alcoholism, which were significantly more common in the propofol group than DEX, did not significantly worsen postoperative pulmonary complications following multivariate logistic regression analysis, OR 0.709, *P* = 0.548 and OR 0.975, *P* = 0.913 respectively.

**Table 4:** Factors associated with postoperative pulmonary complications

Factors	OR	95%*CI*	*P* value
Cardiac arrhythmia	0.70	0.231-	0.548
Alcoholism	9	2.178	0.913
Age	0.97	0.620-	0.189
BMI	5	1.532	0.561
Time of surgery	1.01	0.993-	0.951
Length of stay in hospital	5	1.037	0.399
Duration of mechanical ventilation	1.020	0.953-1.092	0.520
Length of stay in ICU after surgery	1.000	0.992-1.009	0.819
Dex treatment^a^	0.990	0.966-1.014	0.002
CPB assistance	1.003	0.993-1.013	<0.001
	0.999	0.995-1.004	
	0.544	0.368-0.802	
	0.140	0.088-0.223	
Diabetes	1.500	1.018-2.211	0.040
Wound infection	3.995	2.114-7.551	<0.001

^a^Propofol group was the control group; Other variables were negative for the control group

CPB assistance: Extracorporeal circulation used to maintain the stability of circulation, without arresting the heart

### Secondary outcomes

For the secondary outcomes, perioperative DEX infusion was associated with a significant reduction in mechanical lung ventilation duration, LOS in ICU after surgery and LOS in hospital. The mean time to extubation was 18 (13,25) hours in the DEX vs 21 (16,37) hours in the propofol group (*P* < 0.001). LOS in ICU following surgery was 51 (42,90) hours with DEX compared to 59 (46,94.5) hours in the propofol group (*P* = 0.001). The length of hospital stay was also shorter with DEX compared to the propofol group with 20 (17,24) vs 22 (17,28) days, respectively (*P* < 0.001) (Table 3).

**Fig. 2:**
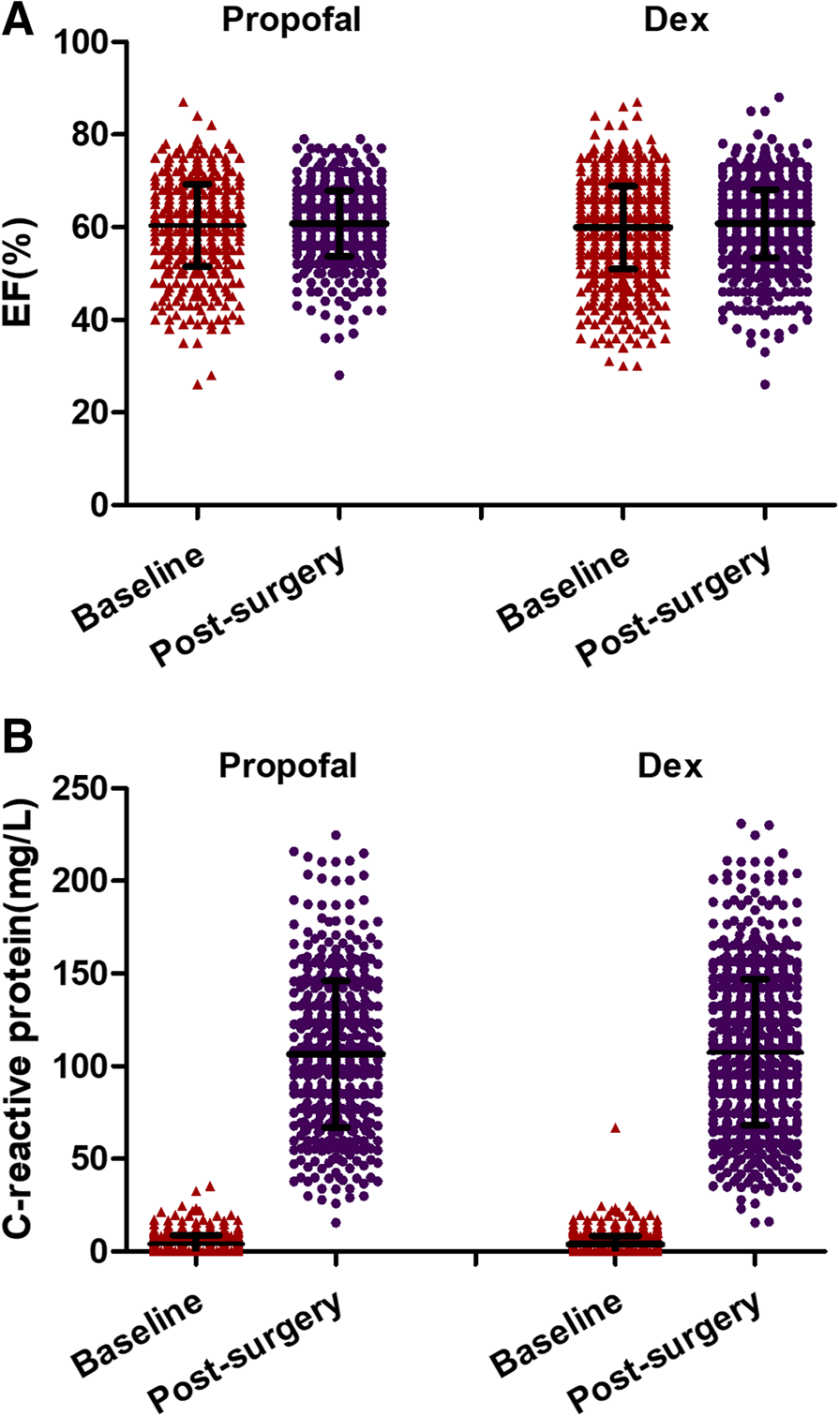
Cardiac ejection fraction (EF) (**A**) and C-reactive protein (CRP) (**B**) data before and after surgery under propofol (*n* = 557) or dexmedetomidine anesthesia (*n* = 831). Data are median (IQR). There was no statistical significance between before and after surgery

The incidence of postoperative wound dehiscence or infection was also significantly lower in the DEX group than propofol; 2.53% vs 6.64%, respectively (*P* < 0.001). There were no significant differences in the 30 days mortality, postoperative complications such as arrhythmias, acute kidney injury, stroke or upper gastrointestinal bleeding (Table 3). Cardiac ejection fraction and C-reactive protein showed no statistical significance between the two groups before and after surgery (Fig. 2).

### Exploratory outcomes

Patients receiving DEX had a slightly shorter surgical time than propofol. Patients in the DEX group required fewer opioids (sufentanil) and inotropic drugs than those in the propofol group (*P* = 0.001). There was no significant difference in the two groups concerning the intraoperative fluid administration, urine output, autologous blood transfusion, extracorporeal circulation, and the use of an intra-aortic balloon pump (Table 5).

**Table 5:** Data during surgery

	Propofol (*n* = 557)	Dexmedetomidine (*n* = 831)	*P* value
Usage of anesthetics			
Sufentanil, μg	271(242,300)	267(233,292)	0.001
Propofol, mg	1105.49(633-2067)		
Dexmedetomidine, μg		217.06(113-593)	
Methoxamine, mg	19(16,21)	13(12,16)	0.001
Time of surgery, min	280(250,320)	275(240,310)	0.005
Intraoperative infusion of crystal fluid, ml	1000(800,1500)	1000(1000,1500)	0.502
Intraoperative infusion of colloidal solution, ml	1000(1000,1500)	1000(1000,1500)	0.564
Intraoperative urine volume, ml	1700(1200,2200)	1700(1200,2300)	0.635
Intraoperative infusion of autologous blood, ml	300(200,500)	300(200,400)	0.164
cardiopulmonary bypass	39(7%)	67(8.06%)	0.466
IABP assistance	27(4.85%)	42(5.05%)	0.862

Data are median (IQR) and patient’s number (%). IABP: Intra-aortic balloon pump

## Discussion

In this large retrospective study of 1388 patients, the perioperative use of DEX shows an overall reduction in postoperative pulmonary complications, duration of mechanical lung ventilation, wound dehiscence or infection, length of ICU and hospital stay. Hypoxemia, atelectasis, pneumonia and bronchospasm are common in patients after cardiac surgery. In this study, these postoperative pulmonary complications tend to be less in the DEX group than in the propofol group, but only atelectasis was statistically significant. Atelectasis could be significantly less in the DEX group due to the reduction of lung inflammation, sputum stasis, and the association with reduction in the ICU mechanical lung ventilation time and is conducive to lung recruitment [14].

DEX has been reported to suppress oxidative stress and inflammatory response in the lung [15] and diminish the severity of acute lung injury produced by remote organ ischemia-reperfusion [16]. The pulmonary protective properties may explain the finding of overall improvement in postoperative pulmonary complications. Our finding was in accordance with a recent meta-analysis of nine randomised controlled trials with a total of 1308 patients. DEX use was also associated with lower incidences of pulmonary complications and less mechanical ventilation time [8]. However, unlike our findings, they did not find any significant differences in other postoperative complications, length of ICU or hospital stay, despite previous systemic review and meta-analysis showing the significant reduction [7]. For 30-day mortality, unlike the previous findings [7], we did not find a significant result for DEX; if anything, DEX was trending towards worse 30-day mortality.

When comparing the preoperative factors to match the propofol and DEX group, we found that arrhythmia and alcoholism were significantly more prevalent in the propofol than in the DEX group. However, upon multivariate logistic regression analysis for postoperative pulmonary complications, we did not find these two factors to worsen postoperative pulmonary complications and therefore did not influence the primary outcome in the propofol group.

We found that patients who had DEX could be extubated three hours earlier than patients who received propofol. These findings fit with DEX’s well-described properties as a compliant, conscious sedative drug that allows more accessible assessment of conscious level, communication between patient and staff, and better pain control [3]. Our study also found the analgesic properties of DEX, where we showed that the group which received DEX required significantly less opioid than the propofol group.

The perioperative administration of DEX has been shown to reduce surgical stress, inflammatory response and preserve the immune cell function following surgery. DEX could significantly reduce the surge of epinephrine, cortisol, interleukin-6 and tumour necrosis factor-α following cardiac surgery [6]. DEX may reduce postoperative complications such as wound dehiscence or infection, as demonstrated in our study, by alleviating excessive surgical stress and inflammatory response [6]. These, together with its cytoprotective effects, might lead to an overall improvement in clinical outcomes.

To the best of our knowledge, our study is the first study to demonstrate that the use of DEX during cardiac surgery may shorten the surgical time compared to propofol. Other studies that compared the duration of surgery between the use of DEX and the controlled group (any treatment without DEX) have not produced a similar finding [17]. However, although not statistically significant, most of these studies were leaning to favour DEX in reducing cardiac surgery duration [17]. DEX has been shown to produce haemodynamic stability with significant beneficial effects on systolic arterial pressure, mean arterial blood pressure, pulmonary artery mean pressure, heart rate and reducing the incidence of hemodynamic complications [9, 17]. These proven benefits may render less intra-operative haemodynamic instability; in particular, slowing down heart rate can enhance better-suturing performance by surgeons and hence reduce surgery time; all of which are useful during cardiac surgery and lead to less surgical time. However, this will need further investigation.

Our study did not find that DEX prevented the occurrence of new onset of cardiac arrhythmias in the postoperative period any more than propofol. This finding is in keeping with those from a recent RCT and observational study [18, 19]. Evidence from other studies remains variable; for example, some studies found the incidence of atrial fibrillation was reduced following the administration of DEX [20, 21].

The cytoprotective effects of DEX on multi-organs have been well documented in the brain, lung, and kidney [21–25]. In oxidative stress-induced lung injury, DEX increased alveolar cell survival and proliferation by activating the protective signalling pathways in lung cells and preventing cellular apoptosis [24]. Both the anti-inflammatory and α_2_ adrenergic receptor-dependent mechanisms provide lung protection against acute lung injury [26]. DEX provides renal protection via the anti-inflammatory effects of the parasympathetic system activation in addition to its direct actions on the α_2_-adrenergic receptor [22]. Serine/threonine-protein kinase, a pathway that plays a crucial role in cytoprotective signalling, is activated by DEX, leading to the reduction in the pathological changes following ischaemia-reperfusion injury in the kidneys. DEX also attenuates Toll-like Receptor 4 (TLR4) expression in tubular cells, which leads to decreased tubular epithelial cell death [23]. All these findings above indicate that DEX may improve short/long term surgical (including cardiac surgeries) outcomes.

Our finding that CPB assistance decreased postoperative pulmonary complications conflicted with previous studies. Multiple inflammatory responses following CPB use has been described as the major causes of pulmonary damage following on-pump CABG surgery [27]. In several studies comparing on-pump and off-pump CABG, CPB used was associated with a significant increase in postoperative pulmonary complications such as pneumonia [28, 29]. Although one study did not find a significant difference in postoperative lung function tests [28]. We used CPB assistance in a specific group of patients with circulatory instability following acute myocardial infarction and heart failure, and therefore our finding cannot be generalized. The correlation between diabetes and postoperative pulmonary complication has also been described as controversial [27]. Although we found that diabetes may worsen postoperative pulmonary complications, another study found no significant difference in pulmonary complications following CABG between patients with or without diabetes [30]. Postoperative factors such as sternotomy infection may negatively affect pulmonary complications [31], as we found.

The strength of this study lies in the inclusion of a large number of patients of a specific type of cardiac CABG surgery, eliminating heterogeneity between different types of cardiac surgery and any potential varied procedural impact on outcomes. However, there are limitations to this study. First, this study is retrospective, which means that there are limitations in interpreting the results for the general patient population or making any concrete conclusions. Second, other postoperative complications were not assessed postoperatively, particularly postoperative delirium, which is prevalent in cardiac surgery. Third, the lung complications between the two groups were marginally different. Lastly, the underlying mechanisms for why DEX reduced postoperative lung complications and enhanced postoperative recovery remain unknown. All warrant further study in the future.

## Conclusions

Our findings suggest that DEX may reduce short term postoperative pulmonary complications, time on mechanical lung ventilation, ICU and hospital stay following CABG surgery compared to propofol. Our work, reported here, may provide a rationale for further prospective clinical studies investigating the benefits of DEX, including other intraoperative outcomes such as the benefit on surgical time or long-term outcomes such as 30 days mortality following CABG surgery.

## Data Availability

The datasets used and/or analysed during the current study are available from the corresponding author on reasonable request.

## Abbreviations

ICU: Intensive care unit
DEX: Dexmedetomidine
CABG: Coronary artery bypass graft
mg: Milligrams
kg: Kilograms
μg: micrograms
I/E: Inspire/expire
VT: Tidal volume
ml: millimetres
min: minutes
hr.: hours
CPB: Cardiopulmonary bypass
LOS: Length of stay
ASA: American Society of Anaesthesiology
SD: Standard deviation
IQR: Interquartile range
TLR4: Toll-like Receptor 4
PaO_2_/FiO_2_: Oxygenation index
PaO_2_: Arterial partial pressure of oxygen
FiO_2_: Inhaled oxygen concentration
PEEP: Positive end-expiratory pressure
